# Fabrication of an Optical Waveguide-Mode-Field Compressor in Glass Using a Femtosecond Laser

**DOI:** 10.3390/ma11101926

**Published:** 2018-10-10

**Authors:** Zhengming Liu, Yang Liao, Zhenhua Wang, Zhihao Zhang, Zhaoxiang Liu, Lingling Qiao, Ya Cheng

**Affiliations:** 1 State Key Laboratory of High Field Laser Physics, Shanghai Institute of Optics and Fine Mechanics, Chinese Academy of Sciences, Shanghai 201800, China; liuzhm@siom.ac.cn (Z.L.); superliao@vip.sina.com (Y.L.); zhangzh94@163.com (Z.Z.); liuzhaoxiang@siom.ac.cn (Z.L.); llq198477@126.com (L.Q.); 2 University of Chinese Academy of Sciences, Beijing 100049, China; 3 School of Physical Science and Technology, Shanghai Tech University, Shanghai 200031, China; 4 State Key Laboratory of Precision Spectroscopy, East China Normal University, Shanghai 200062, China; 5 XXL—The Extreme Optoelectromechanics Laboratory, School of Physics and Materials Science, East China Normal University, Shanghai 200241, China; 6 Collaborative Innovation Center of Extreme Optics, Shanxi University, Taiyuan 030006, China

**Keywords:** optical waveguide, femtosecond laser direct writing, mode-field compressor

## Abstract

We report on fabrication of an optical waveguide-mode-field compressor in glass using a femtosecond laser. Our approach is based on building up a stress field within the waveguiding area which is realized by sandwiching the waveguide between a pair of laser-induced-modification-tracks. To induce an adiabatic conversion of the optical mode in the waveguide, the tracks are intentionally designed to be tapered along the waveguide. We show that our technique can allow for reducing the mode field size in a single mode waveguide from more than 10 μm to around 7 μm.

## 1. Introduction

A femtosecond laser has been used for writing optical waveguides in various types of materials such as glass, crystals, semiconductors and polymers [1,2,3,4,5,6,7,8,9]. The waveguides are produced by space-selectively modifying the refractive index in the laser-irradiated areas which has enabled to produce either type I waveguides by inducing positive refractive changes to form the waveguide cores [1,2,3] or type II waveguides by inducing negative refractive changes to form the waveguide claddings [10,11]. The waveguides are written with various laser parameters and focal conditions to have different propagation modes and/or mode field sizes due to the versatile requirements from the applications [12,13,14,15,16,17,18,19]. Generally speaking, writing optical waveguides in transparent materials with femtosecond laser pulses provides extreme flexibility in terms of the choice of substrate materials, the geometry of the mode field profile, and the configuration of three-dimensional (3D) optical circuits [3,8,20,21,22,23].

One difficulty in the application of laser-written waveguides is to connect the waveguides with external photonic components such as optical fibers or waveguides produced with other technologies. For this purpose, modification of the mode field profiles of the laser-written waveguides is frequently required which can be realized by gradually tuning the laser parameters during the waveguide writing process [21,24]. Nevertheless, since the dependence of the mode field profile on the laser parameters is highly nonlinear, the dynamic tuning of mode field profile typically requires sophisticated beam shaping techniques [24]. Here, we provide an alternative solution to achieve smooth mode field compression in the waveguides written in glass. Our technique is based on a recent finding [25] that stress can be induced in the waveguides by writing two modification tracks in proximity to the waveguides using femtosecond laser direct writing. The stress can promote the refractive index change in the waveguide which gives rise to a significant reduction of the bend loss in the curved portion of the waveguide [25]. Below we show that the stress can also help achieve mode field compression by adiabatically increasing the refractive index along the modification tracks. Our technique can be used for realizing low loss optical connection between the laser written waveguides and external optical networks.

## 2. Materials and Methods

In this work, both the waveguides and the modification tracks were fabricated using a home-built femtosecond laser direct writing setup [18,19,25]. A Ti: sapphire regenerative amplifier (LibraHE, Coherent Inc., Santa Clara, CA, USA) with an operation wavelength of 800 nm, a pulse width of 50 fs, and a repetition rate of 1 kHz was used as the writing laser source. The linearly polarized laser beam was focused into polished fused silica glass (Corning 7979 0F, Corning, NY, USA, 10 mm × 5 mm × 2 mm) using an objective lens with collars that enable spherical aberration correction at different depths in the sample (LUCPLFLN 60×, NA 0.7, Olympus, Tokyo, Japan). The sample was mounted on a computer-controlled XYZ stage for three-dimensional scanning with a translation resolution of 1 μm. 

**Figure 1:**
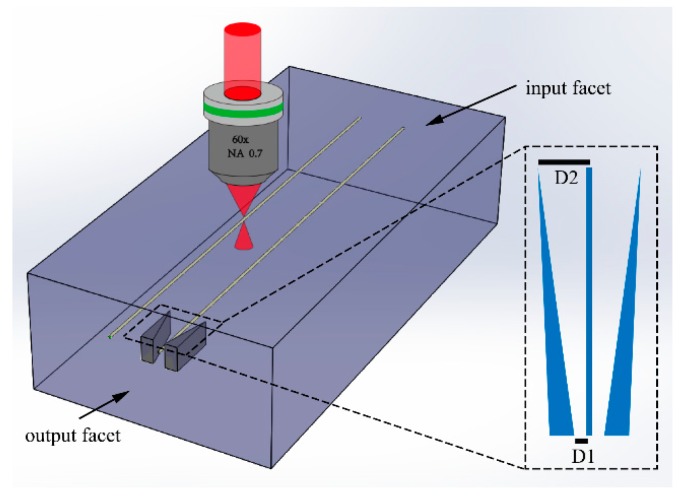
3D sketch of the femtosecond-laser direct-writing waveguides and the tapered modification structures. Inset: top view of the waveguide and tapered structure. D1 and D2: distances between the modification structure and the waveguide at the wider and the narrower ends of the taper respectively.

The waveguides were fabricated along the 5 mm edge of the glass plate with the transverse writing scheme, i.e., the scan direction was perpendicular to the laser propagation direction as illustrated in Figure 1. To obtain a symmetrical cross section, slit beam shaping technique [16] was employed using a phase only spatial light modulator (SLM, X10468-02, Hamamatsu, Japan). The width and length of slit were set at 0.24 mm and 2 mm respectively with the slit orientation parallel to translation direction. Then the slit-shaped beam was mapped onto the pupil plane of the objective lens to write the waveguide 150 μm beneath the top surface of the sample by one single scan with a pulse energy of 1.21 μJ (corresponds to a peak intensity of about 3.8 × 10^13^ W/cm^2^) and a scan speed of 0.01 mm/s.

To achieve mode-field compression, two modification tapers were symmetrically constructed on both side of the waveguide as shown in Figure 1 using matrix writing method. Each layer of the taper was formed by writing three modification lines with a lateral offset of 3 μm at one end of greater width and zero offset at the other end of narrower width. Then five layers of these modification lines were stacked together with an offset of 4.5 μm and a total thickness of about 18 μm in the longitudinal direction to construct the modification taper. The length of the tapers along the waveguide was about 3 mm. The center-to-center distance between the narrower end of the taper and the waveguide was fixed at 15 μm (D2 in Figure 1), which was far enough to avoid the formation of stress field in the waveguide. The distance between the wider end of the taper and the waveguide (D1 in Figure 1) was varied from 3 μm to 6 μm with a step size of 1 μm to test its influence on the mode field compression. Note that the tapers were written without slit shaping, i.e., using a circular input beam of 5 mm in diameter, and the writing pulse energy was about 1.05 μJ (corresponds to the peak intensity of about 5.4 × 10^14^ W/cm^2^) and the scanning speed was 0.06 mm/s.

After inscription of both the optical waveguides and the tapered structures, the end facets of the sample perpendicular to the waveguides were grounded and polished. A 785 nm semiconductor laser (S1FC780PM, Thorlabs, Newton, NJ, USA) was fiber butt-coupled to the input facets of the waveguides with a single mode polarization-maintaining fiber of 780 nm to qualitatively test the guiding properties of the waveguides. The mode field diameter (MFD) measurements were performed by imaging the near field profile on the output facet of the waveguides onto a CCD camera using an objective (20×, NA 0.4) and analyzed using laser beam profiling software (WinCamD series, DataRay Inc., Bella Vista, CA, USA). A 30 μm marker inscribed on the output surface of the sample provided a calibration for the image pixels and the actual dimensions.

## 3. Results and Discussions

**Figure 2:**
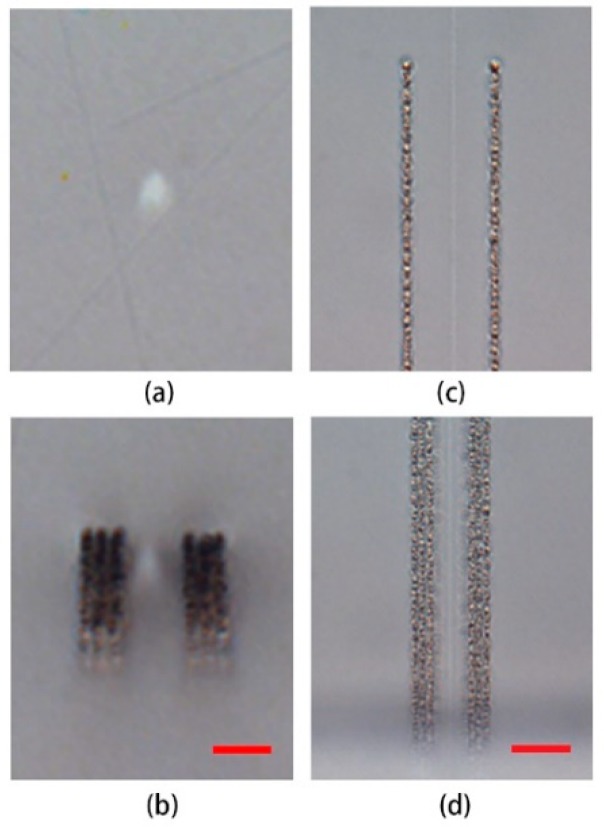
Optical microscopic images of the end-face cross sections of the femtosecond-laser direct-writing waveguides (**a**) without and (**b**) with tapered structure for D1 = 6 μm (scale bar: 10 μm), and the top view microscopic images (**c**) at the narrower end and (**d**) at the wider end of the taper (Scale bar: 20 μm).

The transmission optical microscopy images of the femtosecond laser direct writing waveguides and the tapered mode field modulation structures with D1 = 6 μm are shown in Figure 2, where Figure 2a,b are the cross sections of the waveguides without and with the modification structures, and Figure 2c,d are the top view images at the narrower and the wider ends of the tapered structures respectively. Thanks to the slit shaping technique, the cross sections of the waveguides are nearly circular and the guiding regions appear as the bright spots. Because no slit was used, the laser intensity in writing the tapered structures was higher than the damage threshold of the glass despite that the pulse energy was lower than that for writing the waveguides. Damages such as microcracks and microvoids may have been generated in the laser-inscribed tracks [26], thus, the taper structures are opaque in its cross-sectional view (Figure 2b) and appears rough in its top view (Figure 2c,d). It is worth to note that Bessel-like femtosecond laser beams could be utilized to speed up the fabrication of the tapered mode-field compression structures by reducing the inscription layers due to their advantage in processing laser-writing structures with much higher aspect ratio [27]. However, extra beam shaping techniques has to be employed, and the sidelobes of the Bessel beams should be restrained to avoid certain negative effect on the sandwiched waveguide.

**Figure 3:**
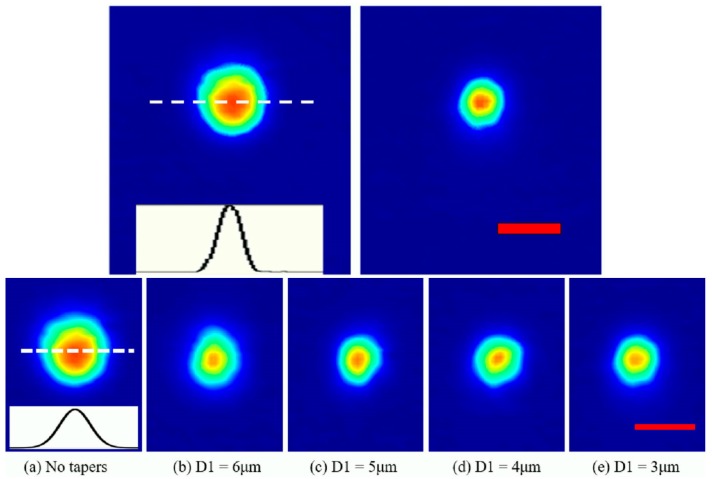
The measured near-field distribution of the waveguides at 785 nm (**a**) without tapers and (**b**–**e**) withthe tapered structures of different D1 at the output facet of the substrate (the inset in (**a**) shows the typical intensity profile at the peak for MFD measurement, scale bar: 10 μm).

The near-field mode intensity distributions of the 785-nm light at the output surfaces of the waveguides without and with the modification structures are shown in Figure 3a–e, respectively. It can be seen that the mode field with the modification structures was strongly compressed, and the mode still maintained the initial Gaussian-like profile. A typical intensity distribution curve of the near-field output mode of the waveguides across the illustrated center section is shown in the inset of Figure 3a, and the MFDs at 1/e^2^ maximum of the waveguides without/with the tapered structures were obtained using Gaussian profile fitting, as listed in Table 1. When there were no tapered structures, the initial waveguide MFD was about 10 μm. When the tapered structures were introduced, the output MFDs is gradually reduced from 7.8 μm to about 7.2 μm with the decrease of D1 from 6 μm to 3 μm, indicating that the compression of mode field relies on the strength of the stress induced in the waveguiding area.

The mechanism of the mode field compression is tentatively given as follows: The femtosecond laser direct writing waveguide in the fused silica glass is a type I waveguide, i.e., a positive refractive index change is induced in the laser-writing line for guiding light, which is resulted from the localized densification and volume reduction in the laser inscribed region [1,28]. As the waveguide is written in the bulk material, the local densification and the volume reduction will induce a tensile force around the waveguide, and the surrounding material will hinder the densification process as well as the increase of the refractive index. The modification tracks written with higher intensity partly destroys the dense Si–O bond of the glass [26,29] and releases the tensile force around the waveguide, which is helpful to the volume shrinkage of the sandwiched waveguide. Meanwhile, the volume of the modification tracks expands slightly due to the generation of molecule oxygen and the formation of microvoids and nanostructures [26,30], which provides additional stress [31,32] and further facilitate the local densification of the waveguide, thereby effective waveguide mode field compression is achieved. Besides, the height of tapered structure is much greater than the waveguide diameter as shown in Figure 2, which can not only release the transverse (left and right) stress beside the waveguides directly, but also release the stress in the upper and lower sides of the waveguides. Therefore, the region above and below the waveguide region will also be subject to the squeeze brought by the tapered structures, giving rise to a rather uniform stress field in both the horizontal and vertical directions. Owing to this effect, the initial Gaussian-like mode profile of the waveguide of a nearly circular cross section is maintained.

Insertion loss is another important parameter for evaluating the performance of waveguides. Although the tapered modification structures appear inhomogeneous and full of scatters as seen in Figure 2, the addition loss due to the introduction of them only occurs when they are much closer to the sandwiched waveguides since the light is mainly guided through the waveguides. The initial insertion loss of the 5 mm waveguide without modification structures was about 1.5 dB due to mode miss-match between the input fiber and the waveguide as well as Fresnel reflection at the end facets etc. By measuring the output powers from the waveguides with (*P*_m_) and without (*P*_0_) the tapered structures using the same input conditions and calculating their ratios, we evaluated the additional insertion loss of the modification structures to the waveguides (*ΔIL*) defined by *ΔIL* = −10lg(*P*_m_/*P*_0_) (dB), as also listed in Table 1. When the distance from the bottom end of the tapered structures to the sandwiched waveguide was larger (D1 = 5 μm and 6 μm), the additional insertion loss was negligible. Thanks to the tapered modification structures, the increase of the refractive index change and the compression of the mode field diameter are changed gradually and smoothly, all the transmitted light field is confined to the waveguide region, thus the mode conversion is performed in an adiabatic way. When D1 was reduced to 4 μm and 3 μm, the additional insertion loss was measured to be about 0.15 dB and 0.32 dB, respectively. This is because when the wider ends of the tapers are very closer to the waveguide, the evanescent field of the guided mode around the waveguide will be coupled into the tapers to induce an additional loss [32]. Since the compressed mode field diameter decreases with the reduction of the separation between the wider end of the tapers to the sandwiched waveguide, while the additional insertion loss changes in opposite way, a tradeoff needs to be made in choosing the appropriate separation distance. In our case, the tapered structure with D1 = 5 μm was the best in our experiments for the balance of mode field compression and additional insertion loss.

## 4. Conclusions

An optical waveguide mode field compressor has been fabricated using femtosecond laser direct writing inside fused silica glass. We achieve an adiabatic conversion of the mode as evidenced by the low addition loss induced by the two modification tracks written in proximity to the waveguide. The mode field size has been reduced from 10 μm to about 7 μm at 785 nm. Since this technique can induce local refractive index modifications in the uniform waveguides written with the fixed laser parameters and focal condition, it opens the possibility of adding extra functionalities in the waveguide such as modifying the mode field profiles and producing Bragg gratings.

## Figures and Tables

**Table 1:** MFD as the function of D1 and the addition loss introduced by the tapered structure.

D1	MFD (μm)	*ΔIL* (dB)
NA (no taper)	10.0	--
6	7.8	NA
5	7.3	NA
4	7.4	0.15
3	7.2	0.32
